# Synthesis and Coordination Behavior of a Flexible Bis(phosphinoferrocene) Ligand

**DOI:** 10.3390/molecules23082054

**Published:** 2018-08-16

**Authors:** Jiří Schulz, Zdeněk Leitner, Ivana Císařová, Petr Štěpnička

**Affiliations:** Department of Inorganic Chemistry, Faculty of Science, Charles University, Hlavova 2030, 128 40 Prague, Czech Republic; jiri.schulz@natur.cuni.cz (J.S.); leitnerz@centrum.cz (Z.L.); ivana.cisarova@natur.cuni.cz (I.C.)

**Keywords:** ferrocene ligands, phosphines, palladium, gold, structure elucidation

## Abstract

A symmetrical flexible bis(phosphinoferrocene) derivative, viz. bis[1′-(diphenylphosphino)ferrocenyl]methane (**1**), was prepared and studied as a ligand in Pd(II) and Au(I) complexes. The reactions of **1** with [PdCl_2_(cod)] (cod = cycloocta-1,5-diene) and [Pd(μ-Cl)(L^NC^)]_2_ (L^NC^ = [2-(dimethylamino-κ*N*)methyl]phenyl-κ*C*^1^) produced bis(phosphine) complex *trans*-[PdCl_2_(**1**-κ^2^P,P′)] (**4**), wherein the ligand spans *trans* positions in the square-planar coordination sphere of Pd(II) and the tetranuclear, P,P-bridged complex [(μ(P,P′)-**1**){PdCl(L^NC^)}_2_] (**5**), respectively. In reactions with the Au(I) precursors [AuCl(tht)] and [Au(tht)_2_][SbF_6_] (tht = tetrahydrothiophene), ligand **1** gave rise to tetranuclear Au_2_Fe_2_ complex [(μ(P,P′)-**1**)(AuCl)_2_] (**6**) and to symmetrical macrocyclic tetramer [Au_4_(μ(P,P′)-**1**)_4_][SbF_6_]_4_ (**7**). All compounds were characterized by spectroscopic methods. In addition, the structures of compound **1**, its synthetic precursor bis[1′-(diphenylphosphino)ferrocenyl]methanone (**3**), and all aforementioned Pd(II) and Au(I) complexes were determined by single-crystal X-ray diffraction analysis (some in solvated form).

## 1. Introduction

**Scheme 1:**
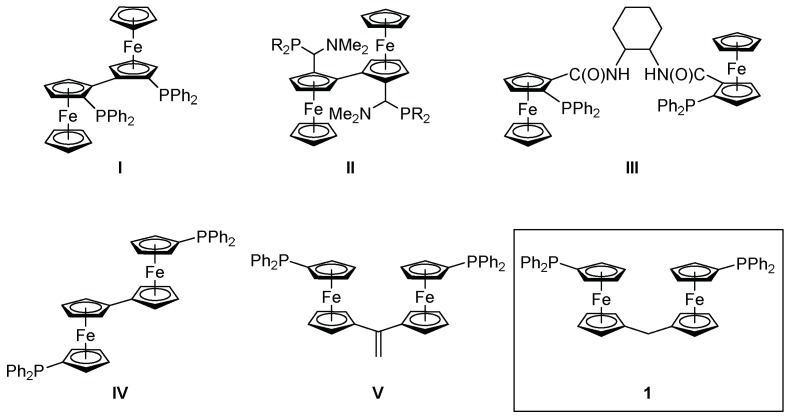
Examples of bis(phosphinoferrocene ligands) and the structure of the newly prepared compound **1**. For clarity, chiral donors are presented without indicating chirality.

Phosphinoferrocene ligands have been extensively studied in recent years, particularly for their numerous applications as versatile supporting ligands in coordination chemistry and catalysis [1,2,3,4]. Although numerous simple and chiral ferrocene phosphines are available, compounds with two or more connected ferrocene units as the molecular scaffold remain less common. Representative examples of chiral bis(phosphinoferrocene) ligands include BIFEP (**I** in Scheme 1) and its analogs with various phosphine substituents [5,6], diphosphines from the TRAP family (**II**) [7,8], and phosphinoferrocenyl analogs of the Trost’s chiral pocket ligand [9] (compound **III** and its stereoisomers) [10,11]. Non-chiral ferrocene phosphines of this type are mainly represented by biferrocene phosphines isomeric to the aforementioned BIFEP ligands, compounds **IV** [12,13]. However, their potentially less structurally confined counterparts, in which two equivalent 1′-(phosphino)ferrocenyl units are connected by one- or two-carbon bridges, are so far represented only by compound **V**. This diphosphine was obtained as a side-product from the reaction of 1′-(diphenylphosphino)-1-acetylferrocene with 1′-(diphenylphosphino)-1-lithioferrocene, and neither its properties nor its coordination behavior have been studied yet [14].

The lack of bis(phosphinoferrocene) ligands with flexible connecting groups led us to design and to prepare a new diphosphine comprising two identical phosphinoferrocene units connected by a methylene linker, viz. bis [1′-(diphenylphosphino)ferrocenyl]methane (**1** in Scheme 1). In this contribution, we describe the synthesis of this compound and its coordination behavior towards Pd(II) and Au(I) ions with various auxiliary ligands in addition to providing a detailed structural characterization (including structure determination) of all compounds prepared.

## 2. Results and Discussion

### 2.1. Synthesis of Ligand ***1***

**Scheme 2:**
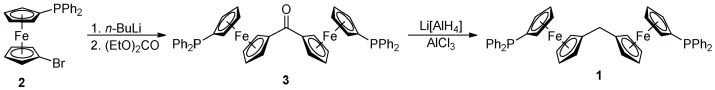
Synthesis of diphosphine **1**.

Diphosphine **1** was prepared in two steps from 1′-(diphenylphosphino)-1-bromoferrocene (compound **2** in Scheme 2). This bromide was lithiated with *n*-butyllithium and the resulting lithio intermediate immediately reacted with diethyl carbonate to give symmetrical ketone **3** in an 88% isolated yield [15,16]. Upon treating with Li[AlH_4_]/AlCl_3_ in tetrahydrofuran [17], this ketone smoothly deoxygenated, thereby producing the target compound **1** as an air-stable, orange solid in a 71% yield, following chromatographic purification and crystallization from hot heptane.

The conversion of **3** into **1** was clearly identified in the NMR spectra, mainly through the replacement of the C=O resonance (δ_C_ 198.61) by signals due to the methylene linker at δ_H_ 2.91 and δ_C_ 28.98. The spectra also indicated the symmetrical nature of **1** and **3**, by showing a single set of signals due to the two chemically equivalent phosphinoferrocene moieties in both cases, and further confirmed that the phosphine moieties remained intact during the synthesis (δ_P_ −16.1 and −17.5 for **1** and **3**, respectively; cf. δ_P_ −18.1 for **2** [18]). Notably, the ν(C=O) band in the IR spectrum of **3** was observed at 1599 cm^−1^, that is, at a lower energy than that of the corresponding band of benzophenone (1666 cm^−1^, Nujol mull [19]) and even of diferrocenyl methanone (1609 cm^−1^ [20]). Both the relatively low δ_C_(C=O) chemical shift and the energy of the ν(C=O) band suggest an extensive conjugation in the molecule of **3**. These findings correspond with the structural data.

**Figure 1:**
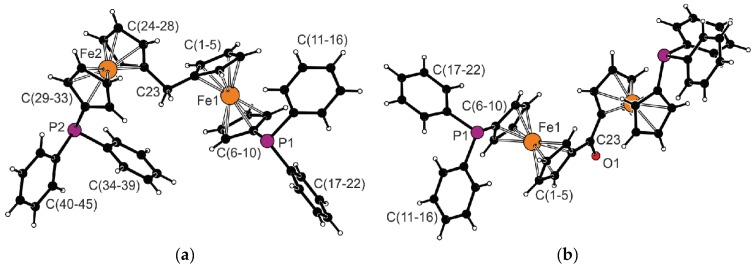
Views of the molecular structures of (**a**) diphosphine **1** and (**b**) ketone **3**; note that the unlabeled half of the molecule of ketone **3** is generated by the crystallographic two-fold axis passing through the C23=O1 bond.

The molecular structures of **1** and **3** determined by single-crystal X-ray diffraction analysis are shown in Figure 1. Pertinent structural parameters are outlined in Table 1. The ferrocene moieties in the structure of diphosphine **1** show regular geometries with similar Fe–C distances (the difference in individual Fe–C distances is smaller than 0.03 Å for an individual Fe atom) and tilt angles well below 5°. However, they differ by the mutual positioning of the substituents bonded at positions 1 and 1′ of the ferrocene core. The substituents in the ferrocene unit comprising atom Fe1 are moved to a farther position than in the other ferrocene fragment (Fe2; compare τ angles in Table 1). The two ferrocene units are mutually rotated, as shown by the dihedral angle of the cyclopentadienyl planes C(1–5) and C(24–28), 80.6(1)°. A similar tilting has been noted for differocenylmethane (82.9(2)°) [20]. The angle at the connecting atom C23, as well as the C1–C23 and C24–C23 distances determined for **1**, fall within the common ranges [21].

The C=O bond in the crystal structure of ketone **3** coincides with the crystallographic two-fold axis (space group *C*2/*c*). Therefore, only the half the molecule is structurally independent. Even in this structure, the ferrocene moieties adopt the usual geometry (Δ(Fe–C) ≈ 0.02 Å) and are negligibly tilted (≈2°). The length of the C=O bond (1.231(3) Å) is identical (within the margins of error) to the C=O bond length determined for diferrocenyl methanone (1.237(3) Å) [22]. The cyclopentadienyl planes connected by the central C=O unit in the molecule of **3** subtend a dihedral angle of 28.7(1)°.

### 2.2. Preparation and Characterization of Pd(II) and Au(I) Complexes

**Scheme 3:**
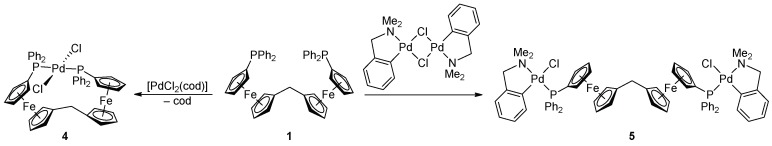
Synthesis of Pd(II) complexes **4** and **5** (cod = cycloocta-1,5-diene).

Two soft transition metal ions were chosen to evaluate the coordination preferences of diphosphine **1**, viz. Pd(II) with a *d*^8^ configuration and a strong preference for the formation of square planar complexes [23] and Au(I) with a more diverse coordination behavior reflecting the lack of crystal field stabilization for a particular coordination arrangement [24]. The palladium precursors used were [PdCl_2_(cod)] (cod = cycloocta-1,5-diene) serving as the source of the PdCl_2_ fragment with two vacant coordination sites, and the dipalladium complex [(L^NC^)Pd(μ-Cl)]_2_ (L^NC^ = 2-[(dimethylamino-κ*N*)mehyl]phenyl-κ*C*^1^) as the source of the (L^NC^)PdCl fragment with only one accessible coordination site and with two adjacent coordination sites blocked by the *ortho*-metallated ligand. The reaction of the former precursor with diphosphine **1** in equimolar amounts led exclusively to red *trans*-chelate complex **1**, whereas the similar reaction of **1** with [(L^NC^)Pd(μ-Cl)]_2_ produced orange tetranuclear Pd_2_Fe_2_ complex **5** (Scheme 3).

The coordination of the phosphine moieties in Pd(II) complexes **4** and **5** was indicated by shifts of their ^31^P-NMR resonances to a lower field. For instance, complex **4** displayed a singlet at δ_P_ 16.0, which is similar to that of the non-chelate analog *trans*-[PdCl_2_(FcPPh_2_-κ*P*)_2_] (Fc = ferrocenyl; δ_P_ 15.8) [25]. In addition, the ^13^C-NMR signals of the ^31^P-coupled carbons (i.e., carbons of the phosphinylated cyclopentadienyl and phenyl rings) were observed as characteristic triplets resulting from virtual coupling in the AXX′ spin system ^13^C-^31^P-M-^31^P-^12^C (M = Pd) [26]. Such a feature is typical of transition metal bis-phosphine complexes. Conversely, compound **5** gave rise to a singlet ^31^P-NMR resonance at δ_P_ 33.0, which corresponds to the position of the signals of [(L^NC^)PdCl(Ph_2_PfcY-κ*P*)_2_] (fc = ferrocene-1,1′-diyl, Y = CH_2_(2-pyridyl): δ_P_ 33.1 in CD_2_Cl_2_ [27], Y = CH_2_OMe: δ_P_ 33.0 in CDCl_3_ [28]) and demonstrates the equivalence of the chemically identical parts of this complex.

**Figure 2:**
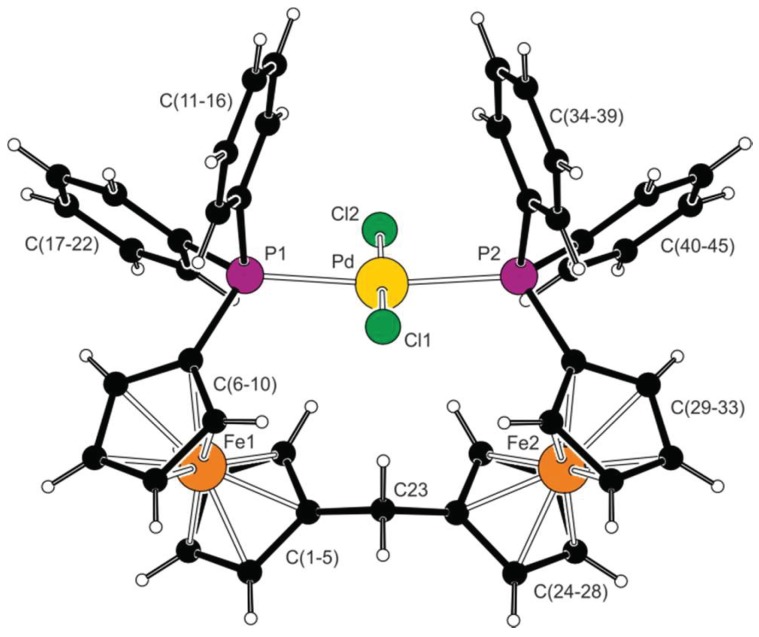
View of the complex molecule in the structure of **4**·CHCl_3_.

Recrystallization of complex **4** from chloroform-hexane provided crystals of the stoichiometric solvate **4**·CHCl_3_, which was structurally characterized by X-ray diffraction analysis. A view of the complex molecule is shown in Figure 2, and the selected geometric parameters are listed in Table 2. The molecule has near-mirror symmetry; however, this is not reflected in the symmetry of the crystal assembly (i.e., the halves of the complex molecule are crystallographically independent but structurally virtually identical) [29]. In such an arrangement, the diphenylphosphino substituents are located on the same side of the diferrocenylmethane scaffold, forming a pocket that accommodates the central atom. The ferrocene units contribute to the lateral placement of the phosphine substituent, adopting a 1,2 or synclinal eclipsed configuration (ideal value: τ = 72° [30]).

The Pd–Cl and Pd–P distances in **4**·CHCl_3_ are similar to those reported for the aforementioned compound *trans*-[PdCl_2_(FcPPh_2_-κ*P*)_2_] [25]. Notably, the two independent Pd–P distances in **4**·CHCl_3_ are almost identical, whereas the Pd–Cl bond lengths differ by nearly 0.03 Å, with the Pd–Cl2 bond directed from the ligand pocket shortened. When viewed along the Cl1⋅⋅⋅Cl2 direction, the palladium atom appears displaced toward the ferrocene units. An alternative view from above the coordination plane reveals an outward displacement for the Pd atom. Consequently, the P1-Pd-P2 angle of 167.49(2)° substantially differs from the ideal 180°, whereas the Cl1-Pd-Cl2 angle is considerably less affected (174.79(2)°). The distortion of the coordination sphere is already indicated by the τ(4) parameter of the tetracoordinate species, being 0.14. The ideal square planar and tetrahedral arrangements would be τ(4) = 0.00 and 1.00, respectively [31].

**Scheme 4:**
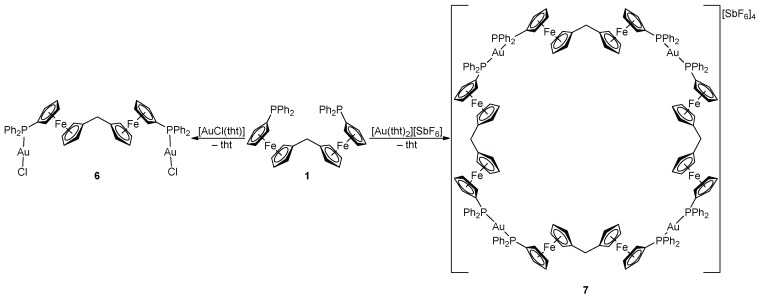
Synthesis of Au(I) complexes **6** and **7** (tht = tetrahydrothiophene).

The reaction of ligand **1** with [AuCl(tht)] (tht = tetrahydrothiophene) at the **1**:Au molar ratio of 1:2 produced the anticipated neutral digold complex **6** as a yellow solid (Scheme 4). When the amount of gold precursor was decreased to 1 equiv., another product was formed. Unfortunately, this product could not be unequivocally characterized due to its poor solubility and reluctance to crystallize. Changing the gold precursor to [Au(tht)_2_][SbF_6_], which lacks a firmly bound halide ligand, the reaction at a 1:1 **1**:Au molar ratio led to the unexpected, cationic macrocyclic tetragold complex **7** (Scheme 4). Compound **7** is cleanly and reproducibly formed and can be isolated in solvated form as a yellow solid in a high yield (93%) by precipitation. The preferential formation of tetrameric **7** over other possible oligomers may be explained by the tetrameric assembly leading to a favorable linear geometry at the gold centers, and possibly by the template effect of the hexafluoroantimonate(V) counter ions also (see the crystal structure below).

The ^1^H- and ^13^C-NMR spectra of **6** show the signals of the diphosphine ligand at the expected position, whereas the ^31^P resonance is characteristically shifted to a lower field (δ_P_ 29.3 in CDCl_3_, cf. δ_P_ 27.6 for [AuCl(FcPPh_2_-κ*P*)] in the same solvent [32]). The formulation of **6** is supported by ESI mass spectra showing monocationic fragments attributable to [M – Cl]^+^ at *m*/*z* 1181. In the case of **7**, the ^1^H- and ^13^C-NMR spectra also display all expected signals of the equivalent ferrocene ligands. The ^13^C-NMR resonances of phosphorus-coupled carbons appear as characteristic virtual triplets (similar to **4**), which further corroborates the formulation of **7** as a symmetrical bis(phosphine) complex. The ^31^P-NMR signal of **7** appears further shifted to a lower field (δ_P_ 40.5 in CD_2_Cl_2_), that is, to the position reported for [Au(Ph_2_PfcSPh-κ*P*)_2_]ClO_4_ (δ_P_ 40.1) [33]. The ESI mass spectrum of **7** showed fragment ions at *m*/*z* 4501 (monoisotopic mass) attributable to the cations [Au_4_(**1**)_4_(SbF_6_)_3_]^+^, thus suggesting that the tetrameric structure of **7** is retained even in solution (at least partly).

**Figure 3:**
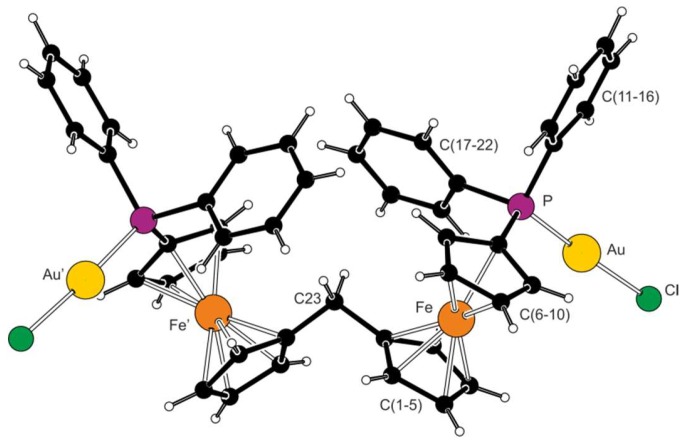
View of the molecular structure of complex **6**; prime-labeled atoms are generated by crystallographic symmetry.

The solid-state structures of both Au(I) complexes were determined by single-crystal X-ray diffraction analysis. The molecular structure of complex **6** is shown in Figure 3, and the relevant structural parameters are outlined in Table 3. In the crystal state, the molecules of the complex reside on the crystallographic two-fold axes passing through the connecting carbon atom C23. The imposed symmetry results in an antiparallel arrangement of the equivalent halves of the complex molecule and the P–Au–Cl subunits (as opposed to the complex molecule in **4**·CHCl_3_, which adopts a near-mirror symmetry).

The Au–Cl and Au–P distances in **6** do not differ from those previously reported for the monogold complex [AuCl(FcPPh_2_-κ*P*)] (2.280(2) and 2.228(2) Å, respectively [32]) and the Au-donor bonds are arranged to form a virtually linear P−Au−Cl fragment (≈176°). The two phosphine moieties and the P−Au−Cl fragments are directed toward opposite sides of the central differocenylmethane unit, and their P−C bonds subtend an angle of 110.53(2)°. The ferrocene cyclopentadienyls assume a near-synclinal eclipsed conformation and are tilted by 3.5(2)°. The P–C bonds in **6** are slightly shorter than in free **1.** These bonds are apparently strengthened by the donation of the phosphorus lone pair and by the associated electron density shift from the conjugated substituents toward the phosphorus atom.

**Figure 4:**
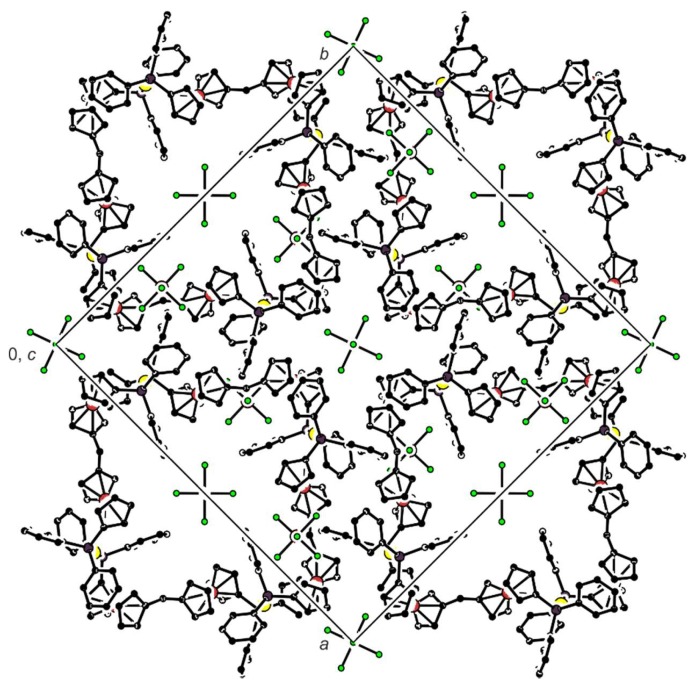
Simplified packing diagram of **7** as viewed along the crystallographic *c* axis; Hydrogen atoms are omitted for clarity. Note that the disordered solvent is not shown.

**Figure 5:**
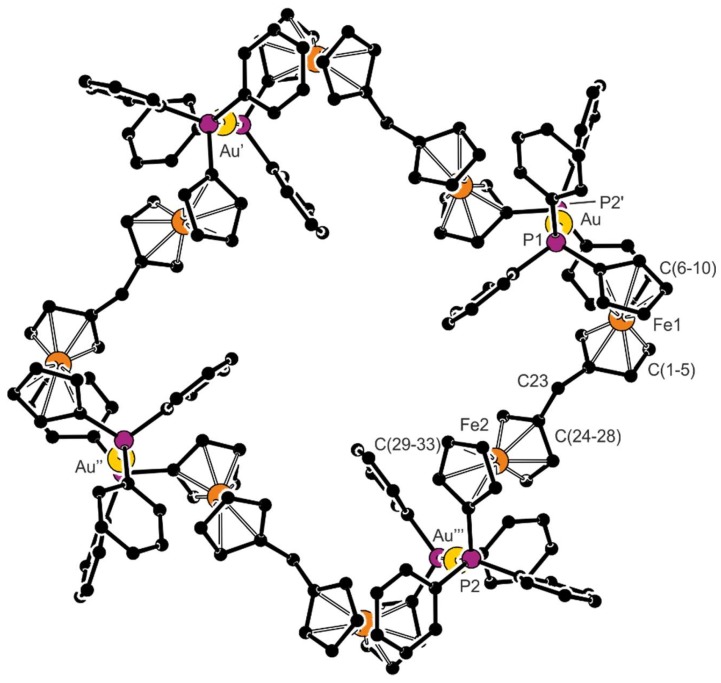
View of the complex cation in the structure of complex **7**; prime-labeled atoms are generated by crystallographic symmetry.

Compound **7** crystallizes in solvated form, but the crystallization solvents are severely disordered within very large, columnar structural voids. Nonetheless, the overall symmetry of the crystal assembly is rather high (tetragonal, space group *I*4/*m*). The complex cations lie over a crystallographic four-fold inversion axis; hence, only one Au(**1**)^+^ subunit is symmetrically independent. The stacking of the complex cations results in the formation of channels oriented along the crystallographic axis *c*, which accommodate the counter anions and the solvent molecules (see the packing diagram in Figure 4). A view of the tetragold cation in the structure of **7** is shown in Figure 5. Selected geometric parameters are presented in Table 4.

The Au–P bonds in **7** are significantly longer than in **6** (by approximately 0.08 Å) but similar to those determined for the discrete bis(phoshine) complex [Au_2_(Ph_2_PfcCN-κ*P*)_2_][SbF_6_]_2_ (Au–P: 2.3104(8) and 2.3140(8) Å) [34]. Qualitatively similar differences can also be detected in the pair of triphenylphosphine complexes, [AuCl(PPh_3_)] (2.228(1) Å) [35] and [Au(PPh_3_)_2_][SbF_6_] (2.319 and 2.324 Å) [36], and are consistent with a larger *trans* influence [37] of the phosphine ligands that destabilize each other. Similarly to **6**, the P−C bonds in **7** are shorter than in the free ligand **1**.

Although the ferrocene cyclopentadienyls in the two structurally independent ferrocene moieties (comprising Fe1 and Fe2) are both almost perfectly eclipsed, they differ in conformation. In the first ferrocene moiety (Fe1), the substituents in positions 1 and 1′ adopt a synclinal eclipsed (or 1,2) conformation. Conversely, in the other (Fe2), they are rotated to a more distant position assuming an anticlinal eclipsed (or 1,3) conformation.

## 3. Experimental

### 3.1. Materials and Methods

All syntheses were performed under an argon atmosphere using standard glassware. Compound **2** [18], [AuCl(tht)] [38], and [Au(tht)_2_][SbF_6_] [34] were prepared according to the literature methods. All other chemicals were purchased from commercial suppliers (Sigma-Aldrich, Alfa-Aesar, UK) and were used as received. Tetrahydrofuran (THF) and dichloromethane were dried with a PureSolv MD5 solvent purification system (Innovative Technology, Inc., Amesbury, MA, USA). Solvents were used without any additional purification during workup, chromatography, and crystallizations (Lach-Ner, Neratovice, Czech Republic).

The NMR spectra were recorded at 25 °C on a Varian UNITY Inova 400 spectrometer (Palo Alto, CA, USA) operating at 399.95 MHz, 100.58 MHz, and 161.90 MHz for ^1^H, ^13^C, and ^31^P, respectively. Chemical shifts (δ in ppm) are given relative to internal tetramethylsilane (^1^H and ^13^C) or to external 85% H_3_PO_4_ (^31^P). In addition to the standard notation of signal multiplicity (s = singlet, d = doublet, t = triplet, etc.), vt and vq are used to denote virtual multiplets arising from the AA′BB′ and AA′BB′X spin systems (A, B = ^1^H, X = ^31^P) constituted by the protons in the C- and Ph_2_P-substituted cyclopentadienyl rings, respectively. FTIR spectra were measured on a Thermo Fisher Nicolet 6700 spectrometer (Waltham, MA, USA) in the range of 400–4000 cm^−1^. Electrospray ionization (ESI) mass spectra were recorded using a Compact QTOF-MS spectrometer (Bruker Daltonics, Billerica, MA, USA). Elemental analyses were performed using a Perkin-Elmer PE 2400 CHN analyzer (Waltham, MA, USA). The amount of residual clathrated solvent (if any) was verified by NMR spectroscopy.

### 3.2. Syntheses

#### 3.2.1. Synthesis of **3**

1′-(Diphenylphosphino)-1-bromoferrocene (**2**; 2.25 g, 5.0 mmol) was dissolved in anhydrous THF (50 mL) in a reaction flask equipped with a stirring bar. The solution was cooled to −78 °C in a dry ice/ethanol bath and treated with *n*-butyllithium (2.0 mL of 2.5 M in hexanes, 5.0 mmol). The resulting mixture was stirred at −78 °C for 20 min before adding neat diethyl carbonate (0.30 mL, 2.5 mmol). The cooling bath was removed, continuously stirring at room temperature overnight. Next, the reaction was terminated by adding saturated aqueous NaHCO_3_ (40 mL), and the mixture was extracted with methyl *tert*-butyl ketone (40 mL). The organic layer was separated and washed with saturated aqueous NaHCO_3_ (40 mL) and brine (2 × 40 mL). The partly separated product was redissolved by adding dichloromethane (50 mL). The organic phase was dried over MgSO_4_ and evaporated, leaving a crude product, which was purified by chromatography over a silica gel column using dichloromethane-methanol (100:1, *v/v*) as the eluent. The methanol content in the mobile phase was gradually increased up to 75:1 (*v/v*), which formed three bands. The two first, minor orange bands, containing non-polar side-products and impurities, were discarded; the third, a major red band, was collected and evaporated to isolate ketone **3** as a red solid. Yield: 1.68 g (88%). Single crystals used for structure determination were grown from warm ethyl acetate.

^1^H-NMR (CDCl_3_): δ 4.09 (vq, *J*′ = 1.8 Hz, 4H), 4.34 (vt, *J*′ = 1.7 Hz, 4H), 4.36 (vt, *J*′ = 2.0 Hz, 4H), 4.80 (vt, *J*′ = 2.0 Hz, 4H) (4 × CH of fc); 7.31–7.39 (m, 20 H, CH of PPh_2_). ^13^C{^1^H}-NMR (CDCl_3_): δ 71.39 (s, 4C, CH of fc), 73.16 (s, 4C, CH of fc), 73.40 (d, *J*_PC_ = 3 Hz, 4C, CH of fc), 74.31 (d, *J*_PC_ = 14 Hz, 4C, CH of fc), 77.91 (d, ^1^*J*_PC_ = 9 Hz, 2C, C_ipso_–P of fc), 80.58 (s, 2C, *C*_ipso_–CO of fc), 128.25 (d, *J*_PC_ = 7 Hz, 8C, CH of PPh_2_), 128.71 (s, 4C, CH of PPh_2_), 133.47 (d, *J*_PC_ = 20 Hz, 8 C, CH of PPh_2_), 138.47 (d, ^1^*J*_PC_ = 10 Hz, 4 C, C_ipso_–P of PPh_2_), 198.61 (s, 1 C, C=O). ^31^P{^1^H}-NMR (CDCl_3_): δ −17.5 (s). IR (Nujol): ν_max_ 1599 s, 1569 w, 1543 w, 1479 m, 1432 s, 1375 s, 1327 w, 1309 m, 1291 s, 1197 m, 1094 w, 1067 m, 1059 m, 1051 m, 1027 m, 998 w, 890 w, 832 s, 810 m, 772 s, 743 s, 699 s, 637 w, 599 w, 499 s, 480 s, 463 m, 447 m, 406 w cm^−1^. ESI + MS: *m*/*z* 767.1 ([M + H]^+^). Anal. Calc. for C_45_H_36_Fe_2_OP_2_ (766.4) C 70.52, H 4.73%. Found: C 70.17, H 4.55%.

#### 3.2.2. Synthesis of Diphosphine **1**

Ketone **3** (1.68 g, 2.2 mmol) was dissolved in anhydrous THF (120 mL) in a reaction flask equipped with a gas inlet, a septum, and a stirring bar. Solid Li[AlH_4_] (0.33 g, 8.8 mmol) was added, and the mixture was stirred at room temperature for 30 min. Then, anhydrous AlCl_3_ was introduced (1.17 g, 8.8 mmol), and the resultant mixture was stirred overnight. The reaction mixture was quenched by carefully adding saturated aqueous NaHCO_3_ (50 mL) and diluting with ethyl acetate (50 mL). The organic layer was separated and consecutively washed with saturated aqueous NaHCO_3_ (2 × 50 mL) and brine (50 mL). The aqueous layers were combined and back-extracted with ethyl acetate (50 mL). The combined organic layers were dried over MgSO_4_ and evaporated. The crude product was taken up with a little dichloromethane and transferred to the top of a silica gel column. Elution with hexane-ethyl acetate (5:1, *v/v*) provided a single orange band (side-products remained adsorbed at the top of the column), which was collected and evaporated. The residue was dissolved in hot ethyl acetate (50 mL), and the solution was poured into hot heptane (30 mL). The crystalline product, which separated upon cooling to 4 °C, was isolated by suction to give pure **1** as an orange crystalline solid. Yield: 1.17 g (71%). Single crystals were grown btained from hot heptane.

^1^H-NMR (CDCl_3_): δ 2.91 (s, 2H, CH_2_), 3.89–3.94 (m, 8H), 4.01 (vq, *J*′ = 1.9 Hz, 4H, CH of fc), 4.27 (vt, *J*′ = 1.8 Hz, 4H, CH of fc); 7.30–7.41 (m, 20H, CH of PPh_2_). ^13^C{^1^H}-NMR (CDCl_3_): δ 28.98 (s, 1C, CH_2_), 68.53 (s, 4C, CH of fc), 69.36 (s, 4C, CH of fc), 75.13 (d, *J*_PC_ = 4 Hz, 4C, CH of fc), 73.47 (d, *J*_PC_ = 15 Hz, 4C, CH of fc), 75.61 (d, ^1^*J*_PC_ = 5 Hz, 2C, C_ipso_–P of fc), 88.91 (s, 2C, *C*_ipso_–CH_2_ of fc), 128.09 (d, *J*_PC_ = 7 Hz, 8C, CH of PPh_2_), 128.42 (s, 4C, CH of Ph), 133.51 (d, *J*_PC_ = 19 Hz, 8C, CH of PPh_2_), 139.29 (d, ^1^*J*_PC_ = 10 Hz, 4C, C_ipso_–P of PPh_2_). ^31^P{^1^H}-NMR (CDCl_3_): δ −16.1 (s). IR (Nujol): ν_max_ 1583 w, 1568 w, 1433 s, 1324 w, 1310 m, 1292 w, 1276 w, 1221 w, 1193 m, 1095 m, 1068 m, 1035 m, 1025 s, 1000 w, 970 vw, 928 m, 917 w, 889 w, 835 s, 819 s, 756 s, 742 s, 698 vs, 630 m, 584 vw, 529 m, 494 vs, 464 s, 456 m, 440 m cm^−1^. ESI + MS: *m*/*z* 753.1 ([M + H]^+^). Anal. Calc. for C_45_H_38_Fe_2_P_2_ (752.4): C 71.83, H 5.09%. Found C 71.71, 5.14%.

#### 3.2.3. Synthesis of Complex **4**

Ligand **1** (75.2 mg, 0.10 mmol) and [PdCl_2_(cod)] (28.5 mg, 0.10 mmol) were dissolved in dichloromethane (5 mL) in a small reaction flask equipped with a stirring bar. The resulting solution was stirred for 3 h and then evaporated. The residue was dissolved in chloroform and crystallized by adding hexane as a top layer. The crystals formed over several days were filtered off and dried under vacuum. Yield of **4**: 91.7 mg (98%), red needles. Crystals of **4**·CHCl_3_ used for X-ray diffraction analysis were obtained by liquid-phase diffusion of hexane into a chloroform solution.

^1^H-NMR (CDCl_3_, 50 °C): δ 4.14 (vt, *J*′ = 1.8 Hz, 4H), 4.48 (vt, *J*′= 1.8 H, 4H), 4.57 (br s, 4H) (3 × CH of fc); 4.80 (br s, 2H, CH_2_), 4.90 (br s, 4H, CH of fc), 7.29–7.40 (m, 12H, CH of PPh_2_), 7.56–7.64 (m, 8H, CH of PPh_2_). ^13^C{^1^H}-NMR (CDCl_3_): δ 31.60 (s, 1C, CH_2_), 68.39 (s, 4 C, CH of fc), 70.21 (t, *J*′ = 27 Hz, 2C, C_ipso_–P of fc), 71.38 (br s, 4C, CH of fc), 71.75 (br s, 4C, CH of fc), 90.1 (s, 2C, *C*_ipso_–CH_2_ of fc), 127.42 (t, *J*′ = 5 Hz, 8C, CH of PPh_2_), 130.03 (s, 4C, CH of PPh_2_), 131.10 (t, *J*′ = 26 Hz, 4 C, C_ipso_–P of PPh_2_), 134.01 (t, *J*′ = 6 Hz, 8 C, CH of PPh_2_). ^31^P{^1^H}-NMR (CDCl_3_): δ 16.0 (s). ESI+ MS: *m*/*z* 893.0 ([M − Cl]^+^). Anal. Calcd. for C_45_H_38_Cl_2_Fe_2_P_2_Pd (929.7): C 58.13, H 4.12%. Found: C 57.80, H 4.29%.

#### 3.2.4. Synthesis of Complex **5**

Ligand **1** (75.2 mg, 0.10 mmol) and [(L^NC^)Pd(μ-Cl)]_2_ (55.2 mg, 0.10 mmol) were dissolved in dichloromethane (5 mL), and the resulting mixture was stirred for 3 h. Following evaporation under vacuum, the crude product was dissolved in a minimum amount of dichloromethane and precipitated by adding pentane. The formed solid was filtered off and dried under vacuum. Yield of **5**: 95.0 mg (77%), orange amorphous solid.

^1^H-NMR (CDCl_3_): δ 2.87 (d, ^4^*J*_PH_ = 2.7 Hz, 12H, N(CH_3_)_2_), 3.38 (s, 2H, CH_2_), 4.07 (d, ^4^*J*_PH_ = 2.2 Hz, 4H, CH_2_N), 4.24 (d of vt, *J* ≈ 1.0 Hz and 2.0 Hz, 4H, CH of fc), 4.26 (vt, *J*′ = 1.8 Hz, 4H, CH of fc), 4.28 (vq, *J*′ = 1.9 Hz, 4H, CH of fc), 4.45 (vt, *J*′ = 1.9 Hz, 4H, CH of fc), 6.31 (ddd, *J* = 7.6, 6.3, 1.2 Hz, 2H, C_6_H_4_), 6.42 (td, *J* = 7.6, 1.4 Hz, 2H, C_6_H_4_), 6.84 (td, *J* = 7.3, 1.2 Hz, 2H, C_6_H_4_), 7.02 (dd, *J* = 7.4, 1.6 Hz, 2H, C_6_H_4_), 7.29–7.36 (m, 8H, CH of PPh_2_), 7.38–7.43 (m, 4H, CH of PPh_2_), 7.52–7.59 (m, 8H, CH of PPh_2_). ^13^C{^1^H}-NMR (CDCl_3_): δ 29.25 (s, 1C, CH_2_), 50.15 (d, ^3^*J*_PC_ = 3 Hz, 4C, N(CH_3_)_2_), 70.00 (s, 4C, CH of fc), 70.64 (s, 4C, CH of fc), 72.67 (d, *J*_PC_ = 7 Hz, 4C, CH of fc), 72.89 (d, ^1^*J*_PC_ = 60 Hz, 2C, C_ipso_–P of fc), 73.60 (d, ^3^*J*_PC_ = 3 Hz, 2C, CH_2_N), 75.78 (d, *J*_PC_ = 10 Hz, 4C, CH of fc), 89.35 (s, *C*_ipso_–CH_2_ of fc), 122.51 (s, 2C, CH of C_6_H_4_), 123.74 (s, 2C, CH of C_6_H_4_), 124.80 (d, *J*_PC_ = 6 Hz, 2C, CH of C_6_H_4_), 127.79 (d, *J*_PC_ = 11 Hz, 8C, CH of PPh_2_), 131.02 (d, *J*_PC_ = 2 Hz, 4C, CH of PPh_2_), 131.87 (d, ^1^*J*_PC_ = 50 Hz, 4 C, C_ipso_ of PPh_2_), 134.37 (d, *J*_PC_ = 12 Hz, 8 C, CH of PPh_2_), 138.44 (d, *J*_PC_ = 10 Hz, 2C, CH of C_6_H_4_), 148.29 (d, *J*_PC_ = 2 Hz, 2C, C_ipso_ of C_6_H_4_), 151.99 (s, 2C, C_ipso_ of C_6_H_4_). ^31^P{^1^H}-NMR (CDCl_3_): δ 33.0 (s). Anal. Calcd. for C_63_H_62_Cl_2_Fe_2_N_2_P_2_Pd_2_ (1304.5): C 58.00, H 4.79, N 2.15%. Found C 57.67, H 4.76, N 1.92%.

#### 3.2.5. Synthesis of Complex **6**

Ligand **1** (75.2 mg, 0.10 mmol) and [AuCl(tht)] (64.0 mg, 0.20 mmol) were dissolved in dichloromethane (5 mL), and the resulting mixture was stirred for 3 h. Subsequent evaporation afforded crude product, which was triturated successively with diethyl ether and pentane until the characteristic odor of tetrahydrothiophene was no longer perceptible and then dried under vacuum. Yield of **6**·0.25CH_2_Cl_2_: 103 mg (71%), orange amorphous solid. Single crystals were obtained upon recrystallization of the compound from chloroform-hexane.

^1^H-NMR (CDCl_3_): δ 3.25 (s, 2H, CH_2_), 3.97 (vt, *J*′ = 1.8 Hz, 4 H, CH of fc), 4.17 (vt, *J*′ = 1.9 Hz, 4H, CH of fc), 4.23–4.25 (m, 4H, CH of fc), 4.57–4.58 (m, 4H, CH of fc), 7.41–7.52 (m, 12H, CH of PPh_2_), 7.54–7.62 (m, 8H, CH of Ph). ^13^C{^1^H}-NMR (CDCl_3_): δ 29.05 (s, 1C, CH_2_), 68.72 (d, ^1^*J*_PC_ = 74 Hz, 2 C, C_ipso_–P of fc), 69.37 (s, 4C, CH of fc), 70.74 (s, 4C, CH of fc), 73.44 (d, *J*_PC_ = 9 Hz, 4C, CH of fc), 74.34 (d, *J*_PC_ = 14 Hz, 4H, CH of fc), 90.12 (s, 2C, *C*_ipso_–CH_2_ of fc), 128.87 (d, *J*_PC_ = 12 Hz, 8C, CH of Ph), 130.92 (d, ^1^*J*_PC_ = 64 Hz, 4C, C_ipso_–P of PPh_2_), 131.58 (d, *J*_PC_ = 2 Hz, 4H, CH of PPh_2_), 133.53 (d, *J*_PC_ = 14 Hz, 8H, CH of PPh_2_). ^31^P{^1^H}-NMR (CDCl_3_): δ 29.2 (s). ESI + MS: *m*/*z* 1181.0 ([M − Cl]^+^). Anal. Calc. for C_45_H_38_Au_2_Cl_2_Fe_2_P_2_·0.25CH_2_Cl_2_ (1238.5): C 43.88, H 3.13%. Found: C 43.81, H 3.01%.

#### 3.2.6. Synthesis of Complex **7**

Ligand **1** (152 mg, 0.20 mmol) and [Au(tht)_2_][SbF_6_] (123 mg, 0.20 mmol) were dissolved in dry dichloromethane (5 mL) and the resulting orange solution was stirred at room temperature for 3 h. Then, the volatiles were removed under vacuum, and the solid residue was repeatedly triturated with diethyl ether and pentane, consecutively, to remove residual tetrahydrothiophene. The powdery residue was dissolved in a minimum amount of dichloromethane and precipitated by adding an excess of pentane. The separated solid was filtered off and dried under vacuum. Yield of **7**: 221 mg (93%), yellow flakes. Crystals used for structure determination were obtained from 1,2-dichloroethane-methyl *tert*-butyl ketone by liquid-phase diffusion.

^1^H-NMR (CD_2_Cl_2_): δ 4.06 (vt, *J*′ = 1.7 Hz, 16H, CH of fc), 4.31 (s, 16H, CH of fc), 4.36 (s, 24H, CH of fc and CH_2_), 4.78 (s, 16H, CH of fc), 7.54–7.66 (m, 80, CH of PPh_2_).^13^C{^1^H}-NMR (CD_2_Cl_2_): δ 32.80 (s, 4C, CH_2_), 68.31 (t, *J*′ = 35 Hz, 8 C, C_ipso_–PPh_2_), 69.53 (s, 16C, CH of fc), 72.08 (s, 16C, CH of fc), 73.81 (t, *J*_PC_ = 4 Hz, 16C, CH of fc), 75.43 (t, *J*_PC_ = 7 Hz, 16C, CH of fc), 88.96 (s, 8C, *C*_ipso_–CH_2_), 130.00 (t, *J*_PC_ = 6 Hz, 32H, CH of PPh_2_), 130.04 (t, *J*_PC_ = 28 Hz, 16C, C_ipso_–P of PPh_2_; partly obscured by the signal due to CH PPh_2_), 132.86 (s, 16C, CH of PPh_2_), 133.90 (t, *J*_PC_ = 7 Hz, 32C, CH of PPh_2_). ^31^P{^1^H}-NMR (CD_2_Cl_2_): δ 40.5 (s). ESI + MS: *m*/*z* 4501.0 ([M − SbF_6_]^+^). Anal. Calc. for C_180_H_152_Au_4_F_24_Fe_8_P_8_Sb_4_ (4740.5): C 45.60, H 3.23%. Found: C 45.34, H 2.97%.

### 3.3. X-ray Crystallography

The diffraction data *θ*_max_ = 26.0° or 27.5° (data completeness ≥ 99.5%) were collected at 120(2) K or 150(2) K using either a Nonius KappaCCD diffractometer with a Bruker Apex-II image plate detector (compounds **3** and **1**) or a Bruker D8 VENTURE Kappa Duo PHOTON100 diffractometer with a IµS micro-focus X-ray tube (other compounds) equipped with a Cryostream Cooler (Oxford Cryosystems, Oxford, United Kindgom). Mo Kα radiation was used in all cases.

The structures were solved by direct methods (SHELXT) [39] and refined using a full-matrix, least-squares routine based on *F*^2^ with SHELXL-97 [40]. The non-hydrogen atoms were refined with anisotropic displacement parameters. Hydrogen atoms were included in their calculated positions and refined using the “riding model” with *U*_iso_(H) set to a multiple of *U*_eq_ of their bonding carbon atom. The structure of compound **7** contained disordered solvent molecules within the columnar cavities. The contribution of the solvent, which could not be resolved in terms of atomic positions, was modeled as diffuse electron density using PLATON SQUEEZE [41].

Relevant crystallographic data, data collection, and structure refinement parameters are presented in Table 5. All geometric calculations were performed using a recent version of the program PLATON [42], which was also used to prepare the structural diagrams. Complete structural data were deposited with the Cambridge Structural Database (CCDC deposition numbers: 1858205–1858209). These data are available free of charge at http://www.ccdc.cam.ac.uk/conts/retrieving.html (or from the CCDC, 12 Union Road, Cambridge CB2 1EZ, UK; Fax: +44-1223-336-033; E-mail: deposit@ccdc.cam.ac.uk).

## 4. Conclusions

In summary, we have reported a facile synthesis of an achiral bis(phosphinoferrocene) ligand **1**. This compound was fully characterized and further demonstrated to coordinate as a normal terminal ligand coordinating two metal centers (P-monodentate donor) in **5** and in **6**, as a *trans*-chelating ligand in **4**, or as a P,P′-bridging ligand forming an unusual macrocyclic tetragold complex **7**. The crystal structures determined that all reported complexes (except for **5**) demonstrate the flexible nature of ligand **1**, which can easily change conformationally (via tilting of the ferrocene moieties and rotation of their cyclopentadienyl rings) to meet the steric demands of the particular central atom to which it is coordinated.

## Figures and Tables

**Table 1:** Selected distances and angles for compounds **1** and **3** (in Å and deg) ^a^.

Parameter	1 (Fe1)	Parameter	1 (Fe2)	Parameter	3 (Fe1) ^b^
Fe–C	2.030(2)–2.057(2)	Fe–C	2.032 (2)–2.051 (2)	Fe–C	2.028 (2)–2.055 (2)
tilt	4.1 (1)	tilt	1.6 (1)	tilt	2.4 (1)
τ	−88.7 (2)	τ	−67.4 (2)	τ	−162.7 (1)
P1-C6	1.805 (2)	P2-C29	1.805 (2)	P1-C6	1.817 (2)
P1-C11	1.838 (2)	P2-C34	1.824 (2)	P1-C11	1.835 (2)
P1-C17	1.839 (2)	P2-C40	1.838 (2)	P1-C17	1.838 (2)
C1-C23	1.500 (3)	C24-C23	1.511 (3)	C1-C23	1.472 (2)
C1-C23-C24	110.8 (2)	–	–	C1-C23-C1′	122.4 (2)

^a^ Definitions: Fe–C the range of Fe–C distances for the particular ferrocene moiety; τ is the torsion angle C1–Cg^C^–Cg_P_–C6 (or similar), where the Cg^C^ and Cg^P^ denote the centroids of the C- and P-substituted cyclopentadienyl ring, respectively; tilt is the dihedral angle of the least-squares cyclopentadienyl planes surrounding the particular Fe atom. ^b^ Further data: C23–O1 = 1.231(3) Å, C1–C23–O1 = 118.8(1)°.

**Table 2:** Selected geometric data for **4**·CHCl_3_^a^.

Parameter	Value	Parameter	Value
Pd-P1	2.3390 (5)	P1-Pd-Cl1	86.14 (2)
Pd-P2	2.3401 (5)	P1-Pd-Cl2	92.95 (2)
Pd-Cl1	2.3167 (5)	P2-Pd-Cl1	86.09 (2)
Pd-Cl2	2.2906 (5)	P2-Pd-Cl2	93.90 (2)
Fe1-C	2.039 (2)–2.061 (2)	Fe2-C	2.034 (2)–2.058 (2)
tilt (Fe1)	0.71 (9)	tilt (Fe2)	1.68 (9)
τ(Fe1)	71.2 (1)	τ(Fe2)	−71.2 (1)
P1-C6	1.805 (2)	P2-C29	1.806 (2)
P1-C11	1.822 (2)	P2-C34	1.821 (2)
P1-C17	1.827 (2)	P2-C40	1.824 (2)
C1-C23	1.509 (2)	C24-C23	1.507 (2)
Fe1, …, Fe2	6.1653 (6)	C1-C23-C24	113.0 (1)

^a^ The parameters are defined as for the free ligand; see footnote to Table 1.

**Table 3:** Selected geometric parameters for complex **6** ^a^.

Parameter	Value	Parameter	Value
Au–P	2.2277 (7)	P–Au–Cl	176.15 (3)
Au–Cl	2.2881 (7)	Au···Au′	12.2322 (7)
Fe–C	2.029 (3)–2.057 (3)	P-C6	1.781 (3)
tilt (Fe)	3.5 (2)	P-C11	1.819 (3)
τ(Fe)	−77.2 (2)	P-C17	1.815 (2)
Fe···Au	4.4147 (5)	C1-C23	1.502 (3)
Fe⋅⋅⋅Fe′	6.3509 (8)	C1-C23-C1′	109.3 (3)

^a^ The parameters are defined as for the free ligand; see footnote to Table 1.

**Table 4:** Selected geometric parameters for the complex cation in the structure of **7** ^a^.

Parameter	Value	Parameter	Value
Au-P1	2.311 (2)	P1-Au-P2′	174.87 (5)
Au-P2′	2.309 (2)	Au···Au’ ^b^	11.6898 (9)
P1⋅⋅⋅P2	10.890 (2)	Au···Au″ ^c^	16.531 (1)
Fe1-C	2.018 (5)–2.063 (6)	Fe2-C	2.022 (6)–2.056 (5)
tilt (Fe1)	2.2 (3)	tilt (Fe2)	3.7 (4)
τ(Fe1)	−77.3 (4)	τ(Fe2)	−144.4 (4)
P1-C6	1.786 (5)	P2-C29	1.792 (6)
P1-C11	1.822 (5)	P2-C34	1.813 (5)
P1-C17	1.817 (5)	P2-C40	1.805 (5)
C1-C23	1.491 (7)	C23-C24	1.503 (8)

^a^ The parameters are defined as for the free ligand; see footnote to Table 1. Further data: C1-C23-C24 = 109.5(4)°. ^b^ Distances between two adjacent gold centers. ^c^ Diagonal Au···Au distance (see Figure 5).

**Table 5:** Summary of relevant crystallographic data and structure refinement parameters.

Compound	1	3	4·CHCl_3_	6	7
Formula	C_45_H_38_Fe_2_P_2_	C_45_H_36_Fe_2_OP_2_	C_46_H_39_Cl_5_Fe_2_P_2_Pd	C_45_H_38_Au_2_Cl_2_Fe_2_P_2_	C_180_H_152_Au_4_F_24_Fe_8_P_8_Sb_4_
*M*	752.39	766.38	1049.06	1217.22	4740.43
Crystal system	monoclinic	monoclinic	triclinic	monoclinic	tetragonal
Space group	*P*2_1_/*c* (No. 14)	*C*2/*c* (No. 15)	*P*–1 (No. 2)	*C*2/*c* (No. 15)	*I*4/*m* (No. 87)
*T*/K	150 (2)	150 (2)	150 (2)	150 (2)	120(2)
*a*/Å	15.6501 (4)	18.7605 (5)	8.9665 (4)	16.8308 (5)	27.6537 (10)
*b*/Å	13.2675 (3)	6.5050 (1)	13.2550 (6)	8.9762 (3)	27.6537 (10)
*c*/Å	17.2689 (4)	29.1239 (7)	18.5420 (8)	26.5919 (9)	28.6005 (13)
α/deg	90	90	82.774 (2)	90	90
β/deg	91.126 (1)	97.5130 (10)	81.222 (1)	94.7570 (10)	90
γ/deg	90	90	77.846 (1)	90	90
*V*/Å^3^	3585.0 (2)	3523.7 (1)	2119.1 (2)	4003.6 (2)	21,872 (2)
*Z*	4	4	2	4	4
*F*(000)	1560	1584	1056	2328	9184
μ (Mo Kα)/mm^−1^	0.931	0.950	1.520	8.262	3.787
Diffrns collected	28,736	25,843	80,051	31,375	101,295
Independent diffrns	8239	4054	9744	4596	10,972
Observed diffrns ^a^	6050	3492	8989	4430	8548
*R*_int_^b^/%	3.23	3.30	2.53	2.15	4.47
No. of param.	442	227	505	241	524
*R*^c^ obsd diffrns/%	3.62	3.27	2.12	1.67	3.61
*R, wR*^c^ all data/%	5.82, 9.43	3.99, 8.72	2.43, 5.15	1.79, 3.59	5.51, 10.1
Δρ/e Å^−3^	0.48, −0.35	0.95, −0.25	0.65, −0.79	1.16, −0.56	2.29, −3.45

^a^ Diffractions with *I* > 2σ(*I*). ^b^ Definitions: *R*_int_ = Σ|*F*_o_^2^ − *F*_o_^2^(mean)|/Σ*F*_o_^2^, where *F*_o_^2^(mean) denotes the average intensity of symmetry-equivalent diffractions. ^c^ *R* = Σ||*F*_o_| − |*F*_c_||/Σ|*F*_o_|, *wR* = [Σ{*w*(*F*_o_^2^ − *F*_c_^2^)_2_}/Σ*w*(*F*_o_^2^)_2_]^½^.
